# *Rickettsia felis* as Emergent Global Threat for Humans

**DOI:** 10.3201/eid1407.071656

**Published:** 2008-07

**Authors:** Carlos E. Pérez-Osorio, Jorge E. Zavala-Velázquez, Juan José Arias León, Jorge E. Zavala-Castro

**Affiliations:** * Universidad Autónoma de Yucatán, Mérida, México

**Keywords:** *Rickettsia felis*, world distribution, vectors, reservoirs, human cases, synopsis

## Abstract

*Rickettsia felis* is an emergent pathogen belonging to transitional group rickettsiae. First described in 1990, *R. felis* infections have been reported to occur worldwide in fleas, mammals, and humans. Because clinical signs of the illness are similar to those of murine typhus and other febrile illnesses such as dengue, the infection in humans is likely underestimated. *R. felis* has been found throughout the world in several types of ectoparasites; cat fleas appear to be the most common vectors. *R. felis* infection should be considered an emergent threat to human health.

## Abstract 2

The reported incidences of human cases and infected vectors have increased during the past 5 years**.**

*Rickettsia felis* is a member of the genus *Rickettsia*, which comprises intracellular pathogens that produce infections commonly called rickettsioses. Although the genus has no recognized subspecies, rickettsiae have traditionally been subdivided into 2 groups: the spotted fever group (SFG) and the typhus group. Infections produced by these 2 groups are clinically indistinguishable; however, groups can be differentiated by outer membrane protein OmpA (absent in the typhus group) and by vector. SFG members are transmitted by ticks; typhus group members, by fleas and lice (*1*,*2*). More recently, Gillespie et al. (*3*) added to this classification by designating the transitional group of rickettsiae and describing an ancestral group of rickettsiae.

In 1990, Adams et al. described a rickettsia-like organism, which resembled *R. typhi,* in the cytoplasm of midgut cells of a colony of cat fleas (*1*). The new rickettsia received the initial name of ELB agent after the company from which the fleas were obtained (El Labs, Soquel, CA, USA) (*4*). The first observations, such as reactivity with antibodies to *R. typhi* (*1*), the type of vector in which it was first discovered (*1*), and the apparent absence of OmpA (*5*), suggested that the new organism belonged to the typhus group of rickettsiae (*4*).

The molecular characterization of the organism described by Adams and reported by Bouyer et al. in 2001 provided sufficient evidence to support the designation of *R. felis* as a member of the SFG (*6*), and in 2002, La Scola et al. provided further characterization (*7*). One noticeable characteristic is the temperature-dependent growth of the bacterium, which requires incubation temperatures of 28°–32°C for optimal growth. However, the most striking characteristic of the novel rickettsia was the plasmid DNA in its genome (*8*).

## World Distribution in Potential Host Vectors

Soon after the initial description of the typhus-like rickettsia, Williams et al. (*9*) reported that cat fleas collected from opossums in an urban setting in California were infected with the novel rickettsia, but no organism was detected in the tissues of the opossums. Since this report, this organism has been described in infected vectors from 20 countries on 5 continents (*9*). Not until 2002 did interest in *R. felis* increase, when the United States (*9*), Brazil (*10*), Mexico (*11*), and Spain (*12*) were among the first countries to describe cat fleas (*Ctenocephalides felis*) infected with *R. felis.* During the following 5 years, 28 additional reports appeared from all over the world (Table 1). These reports describe new potential vectors being infected with the emergent rickettsia, including the following: fleas, such as *C. canis* (*13*–*15*), *Anomiopsyllus nudata* (*16*), *Archaeopsylla erinacei* (*15*,*17*), *Ctenophthalmus* sp (*17*), and *Xenopsylla cheopis* (*18*); ticks, *Haemaphysalis flava* (*19*), *Rhipicephalus sanguineus* (*20*), and *Ixodes ovatus* (*19*); and mites from South Korea (*21*) (Table 1). Despite the large number of potential vectors reported, the only vector currently recognized is *C. felis* because it has been demonstrated that this flea is able to maintain a stable infected progeny through transovarial transmission (*4*). In addition, production of antibody to *R. felis* has been noted in animals after they have been exposed to infected cat fleas (*9*). Other evidence to be considered is the fact that 68.8% of the reports state that the cat flea is the most recurrent vector in which *R. felis* has been detected. These data further support the wide distribution of rickettsiae because they correlate with the worldwide distribution of *C. felis;* this distribution represents a threat to the human population because of lack of host specificity of the cat flea.

**Table 1:** Potential vectors infected with *Rickettsia felis* reported worldwide, 1992–2007*

Year	Source of DNA sample	Animal†	Country	Reference
1992	*Ctenocephalides felis*	Opossum	USA	(*9*)
2002	*C. felis*	Cats and dogs	Brazil	(*10*)
2002	*C. felis*	Dogs	Mexico	(*11*)
2002	*C.felis*	Cats and dogs	Spain	(*12*)
2003	*Haemophysalis flava, H. kitaokai,* and *Ixodes ovatus*	Unknown (flagging)	Japan	(*19*)
2003	*C. felis*	Cats	France	(*22*)
2003	*C. felis*	Cats and dogs	UK	(*23*)
2004	*C. felis*	Dogs	Peru	(*24*)
2005	*Anomiopsyllus nudata*	Wild rodents	USA	(*16*)
2005	*C. felis*	Cats and dogs	New Zealand	(*25*)
2005	*C. felis*	Monkey	Gabon	(*26*)
2006	*C. felis* and *C. canis*	Dogs	Brazil	(*13*)
2006	*C. felis* and *C. canis*	Cats and dogs	Uruguay	(*14*)
2006	*Archaeopsylla erinacei* and *C. canis*	Hedgehog and rodents	Algeria	(*15*)
2006	*Archaeopsylla erinacei* and *Ctenophtalmus sp.*	Rodents and hedgehog	Portugal	(*17*)
2006	*Xenopsylla cheopis*	Rodents‡	Indonesia	(*18*)
2006	*C. felis, Rhipicephalus sanguineus,* and *Amblyommma cajennense*	Dogs and horse	Brazil	(*20*)
2006	Unknown flea	Gerbil	Afghanistan	(*27*)
2006	*C. felis*	Cats and dogs	Australia	(*28*)
2006	*C. felis*	Cats	Israel	(*29*)
2006	*C. felis*	Rodents	Cyprus	(*30*)
2007	Mites	Wild rodents	Korea	(*21*)
2007	*C. felis*	Cats	USA	(*31*)
2007	*C. felis*	Cats	Chile	(*32*)

*PCR was used to detect *R. felis* infection with 1 noted exception. †Animal host of potential vectors. ‡Quantitative PCR.

*R. felis* infection is diagnosed by PCR amplification of targeted genes. The genes most commonly amplified by researchers are *gltA* and *ompB*; followed by the 17-kDa gene. Also, 25% of published articles report that *R. felis* was detected by amplifying >2 genes, and all report that amplicons were confirmed as *R. felis* by sequencing. The animal hosts from which the infected ectoparasites were recovered represent a diversity of mammals (Table 1), which included 9 different naturally infested animal species. However, in 16 of 33 articles, ectoparasites were recovered from dogs. Other hosts for ectoparasites were cats (in 13 of 33 reports); rodents (5 of 33 reports); opossums and hedgehogs (2 reports each); and horses, sheep, goats, gerbils, and monkeys (1 report for each animal species).

In summary, the presence of *R. felis* in a diverse range of invertebrate and mammalian hosts represents a high potential risk for public health and the need for further studies to establish the role of ectoparasites other than *C. felis* as potential vectors. To date, whether any vertebrate may serve as the reservoir of this emergent pathogen has not been determined. However, preliminary data from our laboratory suggest that opossums are the most likely candidates.

## World Distribution of Human Cases

In 1994, the first human case of infection with the new cat flea rickettsia was reported in the United States (*2*). This became the first evidence of *R. felis’* potential as a human pathogen. *R. felis* infection had a similar clinical manifestation as murine typhus (including high fever [39°–40°C], myalgia, and rash). Although the initial idea was that the murine typhus–like rickettsia had a transmission cycle involving cat fleas and opossums (*2*,*5*,*9*), no viable *R. felis* has yet been isolated from a vertebrate host.

Three more cases of *R. felis* infection were reported from southeastern Mexico in 2000. The patients had had contact with fleas or animals known to carry fleas. The clinical manifestations were those of a typical rickettsiosis: all patients had fever and myalgia; but the skin lesions, instead of a rash, were similar to those described for rickettsialpox. In addition, for 3 patients, central nervous system involvement developed, manifested as photophobia, hearing loss, and signs of meningitis (*33*).

As occurred with the fast-growing reports of the worldwide detection of *R. felis* in arthropod hosts, the reports of human cases of *R. felis* infection increased rapidly in the following years (Table 2). But, in contrast, only 11 articles reported human infection by *R. felis* compared with 32 that reported ectoparasite infection with the new rickettsia. Nevertheless, these findings indicate that an effective surveillance system is urgently needed to distinguish *R. felis* rickettsiosis from other rickettsial infections such as murine typhus and Rocky Mountain spotted fever, and from other febrile illnesses such as dengue. Although PCR is still a method of choice for many laboratories, its high cost prevents many from using the technique, particularly in developing countries. Important advances have been achieved in diagnostics, such as the recent establishment of a stable culture of *R. felis* in cell lines that allows its use as antigen in serologic assays differentiating the cat flea rickettsia from others. Use of this culture in the immunofluorecent assay has enabled detection of additional human cases (*38*).

**Table 2:** Human cases of *Rickettsis felis* infection reported worldwide, 1994–2006*

Year	No. cases	Method	Country	Reference
1994	1	PCR	USA	(*2*)
2000, 2006	5	PCR	Mexico	(*33*)
2001, 2006	3	PCR	Brazil	(*34*)
2002	2	PCR/serology	Germany	(*35*)
2003	1	Serology (seroconversion)	Thailand	(*36*)
2005	3	Serology (Western blot)	South Korea	(*37*)
2006	8	Serology (IFAT/Western blot)	Tunisia	(*38*)
2006	1	Serology (seroconversion)	Laos	(*39*)
2006	33	Serology (IFAT)	Spain	(*40*)
Total	68			

*IFAT, indirect fluorescent antibody test.

The first autochthonous human case in Europe was reported in 2002, which demonstrated that *R. felis* has a potential widespread distribution and is not confined to the Americas. It also confirmed the risk for human disease anywhere in the world (*35*). After the first report in Europe of a human infection of *R. felis*, other human cases have appeared in other countries around the world, including Thailand (*36*), Tunisia (*38*), Laos (*39*), and Spain (*40*); additional cases have been reported in Mexico and Brazil (*34*). All the data support the conclusion that the incidence of *R. felis* rickettsiosis and the simultaneous worldwide distribution of the flea vector plausibly explain its endemicity.

At present, the involvement of domestic animals (e.g., dogs and cats) or wild animals coexisting in urban areas (e.g., opossums) maintains *R. felis* infection in nature. *C. felis* fleas serve as the main reservoir and likely have a central role in transmission of human illness.

## Conclusions

*R. felis* is an emergent rickettsial pathogen with a worldwide distribution in mammals, humans, and ectoparasites. The clinical manifestations of *R. felis* infections resemble those of murine typhus and dengue, which makes them difficult to diagnose without an appropriate laboratory test. For this reason, infections due to this emergent pathogen are likely underestimated and misdiagnosed. Although *R. felis* may require only fleas for its maintenance in nature, we still do not know the role of animals in the life cycle of flea-borne spotted fever rickettsia. In addition, flea-borne spotted fever should be considered in the differential diagnosis of infectious diseases. Further research should be conducted to determine the actual incidence of *R. felis* infection in humans, the spectrum of clinical signs and symptoms, and the severity of this infection and also to assess the impact on public health.
